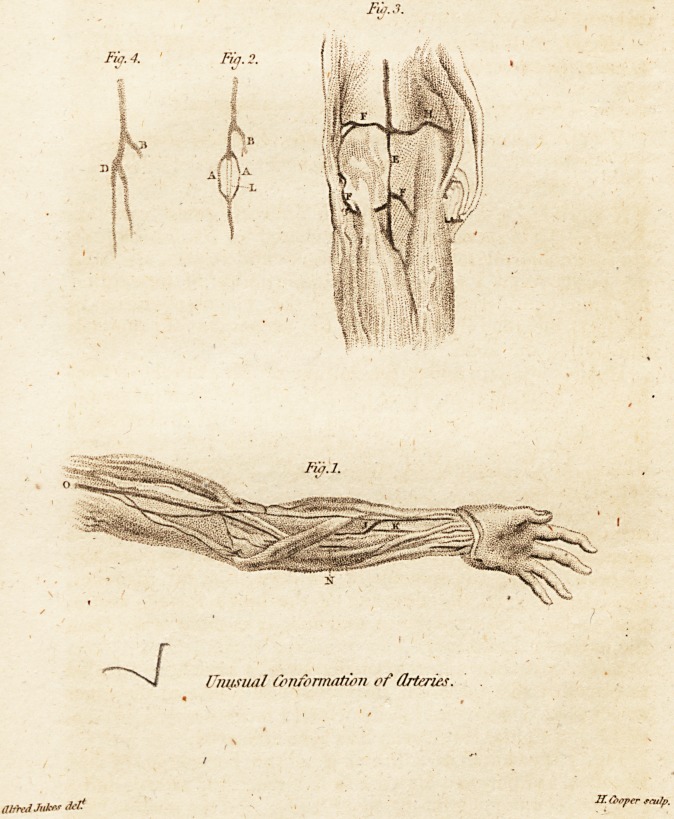# Mr. Jukes on Varieties in the Arterial System

**Published:** 1812-12

**Authors:** Alfred Jukes

**Affiliations:** Birmingham


*To the Editors of the Medical and Physical Journal.*

(With a Plate.)

GENTLEMEN,

IN perusing the last Edinburgh Journal, some very interesting observations, communicated by Dr. Ramsay, on unusual conformations of some muscles and vessels, attracted my notice; and I embrace this opportunity of forwarding for your consideration a few remarks, in corroboration of the facts recorded by him, with the accompanying sketches illustrating the same.

Having studied under the tuition of Mr. Brookes, Professor of Anatomy, in whose rooms it is likewise an invariable rule to have all bodies injected previous to dissection, I have almost daily witnessed varieties in the arterial system, which, in the uninjected state, might, probably, have escaped notice.

The arteria obturatrix (a branch of the hypogastric), mentioned in some late invaluable treatises on Hernia, as occasionally arising from the epigastric, is a fact so generally observed among the students of his class, that Mr. Brookes, when treating on the doctrine of the blood-vessels, states, that, as far as his experience extends, it as often arises from the latter as the former.

In two patients requiring phlebotomy, I have been enabled to trace, by the pulsation, the superficial course of the ulnar artery; and, in several bodies which I have assisted in dissecting, a similar singularity has been observed.

Fig. 1 is a sketch of a drawing in the possession of Mr. Brookes, exhibiting a high division of the radial artery, and a singular communication between it (J); and the median (K), an artery also very frequently found, and perhaps I may say universally, when there is any unusual course of the other arteries of the arm. The fellow extremity had a high division in the axilla.

Fig.

*Fig. 3.*

*Fig. 4.*

*Fig. 2.*

*Fig. 1.*

*Unusual Conformation of Arteries.*

*Alfred Jukes del.t* *H. Cooper sculp.*

Fig. 2 represents an obliteration of the arteria femoralis superficialis. It appeared as if it were converted into a ligamentous substance. (AA) are two branches given off above, and communicating with the continued trunk below.

Fig. 3. A considerable portion of the popliteal artery (E) was contracted in size, yet remaining somewhat pervious. (FF.FF) are the articular arteries enormously dilated. The inosculation with the recurrent branch of the anterior tibial was very large.

Fig. 4 was from the fellow limb of fig. 2, showing a double arteria femoralis superficialis; and, in Mr. Brookes's museum, is a fœtus exhibiting the same conformation.

I have seen two instances of an artery, arising in common with the trunk of the epigastric, as described by Dr. Ramsay. Many other instances, of less practical importance, might be enumerated, clearly pointing out the utility of injecting the bodies prior to dissection; and, where it is much practised, and frequent comparisons between limbs are made, the distribution of the blood-vessels of each will rarely be found to correspond.

With respect, I remain,

*Birmingham.* Your's, &c.

ALFRED JUKES.

*Reference to the Plate.*

N. The ulnar artery.
O. Brachial.
B. Profunda femoris.
L. The obliterated portion.
D. The bifurated superficial femoral artery.

## Figures and Tables

**Fig. 1. Fig. 2. Fig. 3. Fig. 4. f1:**